# Numerical Simulation of Droplets Behavior of Cu-Pb Immiscible Alloys Solidifying under Magnetic Field

**DOI:** 10.3390/ma10091005

**Published:** 2017-08-28

**Authors:** Lin Zhang, Tiannan Man, Minghao Huang, Jianwen Gao, Xiaowei Zuo, Engang Wang

**Affiliations:** Key Laboratory of Electromagnetic Processing of Materials, Ministry of Education, Northeastern University, Shenyang 110004, China; manxiaoxiao165@163.com (T.M.); huangminghao92@126.com (M.H.); xiaomu1218@163.com (J.G.); zuoxw@epm.neu.edu.cn (X.Z.)

**Keywords:** immiscible alloys, coagulation, migration, high magnetic field (HMF), melt flow

## Abstract

A model has been presented for the coarsening of the dispersed phase of liquid-liquid two-phase mixtures in Cu-Pb alloys under the effect of a high magnetic field (HMF). The numerical results show that the evolution of size distribution is the result of several factors and the diffusional growth, the collision-coagulation of the Cu-rich droplets (gravity sedimentation and Marangoni migration), and melt flow also have obvious effects on the movement of droplets and coarsening process. The effect of the HMF in the coarsening process of Cu-Pb alloy is studied in this work both by simulation and experiment. The analysis shows that the HMF leads to a decrease in the melt flow velocity, and can also lead to a decrease in the moving velocity of Cu-rich droplets. The HMF significantly reduces the coarsening rate of droplets as compared by the distribution evolutions. Finally, it is shown that droplet collision and coagulation can be dramatically retarded by the HMF. The results of the simulation are compared with the experiments performed with immiscible Cu-Pb alloys, and the discrepancy between theory and experiment is discussed.

## 1. Introduction

Immiscible alloys exhibit a miscibility gap in the phase diagram, which always caused a serious separation during the solidification process. The liquid coarsening process does not occur instantaneously but, generally, the second phase is first nucleated as very fine droplets, which grow by diffusion and can also move and coagulate due to gravity and interfacial tension leading, finally, to a macroscopic segregation. Immiscible alloys are difficult to fabricate by conventional casting methods, whereas they have good physical and chemical properties, providing the minority phase can be well-dispersed in the solid microstructure. Immiscible alloys, such as Cu-Pb, Al-Pb, or Al-Bi, are potential materials for advanced bearings in automotive applications.

Great improvements in the simulation of liquid-liquid decomposition and spatial separation have been made in recent decades, and most of these works used a classical modeling, named as the population dynamic approach, to simulate the coagulation in immiscible alloys [1,2,3,4,5,6,7,8]. The coarsening process is so complicated that the expression equation cannot be solved analytically or numerically, therefore, the model needs to be simplified to explain the experimental results to a certain degree, and the actual solidifying process is difficult to reflect.

The discrete multi-particle approach (DMPA) has been developed by Ratke and Diefenbach [9] to calculate the phase decomposition and microstructure evolution in immiscible alloys. In this approach the temperature field in a specimen is obtained through measurement and calculation. In as much as the temperature decreased, the droplets are nucleated. The nucleated droplets grow by diffusion, and they are able to move in the temperature field by Marangoni motion and in the gravity field by Stokes motion. During the coarsening process, the position of each droplet must be memorized. Thus, the size distribution of droplets in a cross-section of the specimen is obtained and can be compared with experimental results. Such a numerical simulation can start with a few million virtual droplets, thus, the location and coagulation behavior of each droplet at any moment is difficult to track. Guo et al. [10] simplified this approach by dividing the droplets into various size classes according to their radius. They tracked the various size classes and did not need to track a large number of individual droplets, which greatly reduced the length of the computation.

The results of the simulation with the whole coarsening process depend on the accuracy of thermophysical properties, especially for the nucleation. The nucleation process is complicated under experimental conditions, and the factors, such as impurities and undercooling, affect the nucleation rate considerably, which can lead to a large difference between the theoretical prediction and the experimental results. Rogers and Davis [4] eliminate the need of nucleation by fabricating a base material by rapid cooling, then heating the base material to a temperature within the miscibility gap which would be sufficient to melt both the droplet and matrix phases. The information about the initial droplet population can be inferred from studies of the base material.

We develop a method to simulate the behavior of many droplets in the coarsening process of immiscible alloys, which has the same basic route as the discrete multi-particle approach to track the location, velocity, and other properties of each single droplet. This study focuses on the behavior and spatial distribution of droplets in the later period of the coarsening process, including the diffusional growth, the Stokes motion, and the Marangoni motion.

In some investigations, experimental evidence of the effect of the HMF on the coarsening process of immiscible alloys has been obtained [11,12]. The cooling rate is slow and a complete segregation is formed in the sample without any HMF. However, there are still a large number of dispersive Cu-rich droplets distributed in the Pb-rich matrix under the HMF. With the model in this paper, we investigate the evolution of droplets coarsening under the same cooling rate as an experiment, and investigate the coarsening mechanism of dispersive Cu-rich droplets. The effect of the HMF on the melt flow and droplet motion is also discussed.

Cu-55 at % Pb alloy was used in this research, and the sample size is 10 mm in diameter and 20 mm in height. To compare with the simulation results, a solidification experiment was performed under a 12 T HMF. The direction of the magnetic field is vertically upward. In the solidifying process, the cooling rate of the melt is 0.74 °C/s when its temperature passed through the miscibility gap.

## 2. Mathematical Formulation 

The temperature field and velocity field of the melt flow both depend on time and position. In a cylinder crucible, the melt flow of one of the transverse directions, the θ direction was neglected, and a 2-D flow model has been adopted in this study. An incompressible Newtonian fluid was assumed. The following equations are for 2-D incompressible flow in an axisymmetric system. In the case of an electrically-conducting, incompressible fluid, temperature (*T*), magnetic ($\vec{B}$) and velocity ($\vec{U}$) fields are governed by the following MHD equations:

(1) Continuity equation:

(1) $$\nabla \cdot \vec{U}=0$$

where $\vec{U}$ denotes the velocity.

(2) Momentum equation:

(2) $$\frac{\partial \vec{U}}{\partial t}+(\vec{U}\cdot \nabla )\vec{U}=\eta \nabla^{2}\vec{U}-\frac{1}{\rho }\nabla P+\frac{1}{\rho }{\vec{F}}_{g}+\frac{1}{\rho }{\vec{F}}_{m}$$

where *η* denotes the viscosity, *ρ* denotes the density, ${\vec{F}}_{g}$ denotes gravity, and ${\vec{F}}_{m}$ denotes the Lorentz force caused by a magnetic field with flux density $\vec{B}$:

(3) $${\vec{F}}_{m}=\vec{J}\times \vec{B}=-\nabla \left(\frac{{\vec{B}}^{2}}{2\mu_{m}}\right)+\left(\vec{B}\cdot \nabla \right)\frac{\vec{B}}{\mu_{m}}$$

where *μ_m_* denotes the magnetic permeability.

(3) The equation of the magnetic field is deduced from Maxwell equation and Ohm’s law:

(4) $$\frac{\partial \vec{B}}{\partial t}+\left(\vec{U}\cdot \nabla \right)\vec{B}=\left(\vec{B}\cdot \nabla \right)\vec{U}+\frac{1}{\mu_{m}\sigma }\nabla^{2}\vec{B}$$

where *σ* denotes the electric conductivity.

(4) Temperature equation:

(5) $$\frac{\partial T}{\partial t}+\left(T\cdot \nabla \right)\vec{U}=\frac{\lambda }{\rho c}\nabla^{2}T+\frac{Q\;}{\rho c}$$

where *λ* denotes the thermal conductivity, *c* denotes the specific heat capacity, and *Q* is the energy generated per unit volume. The original temperature of the fluid is set to be 963 °C. As the center of sample, the left wall is assumed to be the adiabatic boundary condition. The side wall, bottom wall, and top wall are assumed to be the Neumann boundary conditions. We assume the surface heat flux to be 312 W·m^−2^ at the bottom and side walls, and 156 W·m^−2^ at the top, respectively. In the solidifying process of comparison experiments, the cooling rate of the melt is 0.74 °C/s when its temperature passes through the miscibility gap. With the boundary conditions assumed above, the average temperature in the simulation is changed similarly with the results in comparison to the experiment measured by a thermocouple.

(5) Concentration Equation:

(6) $$\frac{\partial x_{Cu}}{\partial t}+(x_{Cu}.\nabla )\vec{U}=D_{Cu}\nabla^{2}x_{Cu}+R_{Cu}$$

where *x_Cu_* is the concentration of Cu, *D_Cu_* is the diffusion coefficient of Cu in matrix, *R_Cu_* is a source term and denotes the loss of Cu concentration in the matrix due to the growth of Cu-rich droplets, which is calculated by Equations (14) and (15).

## 3. Droplet Growth Model and Calculations

The main objective of this paper is to investigate the coarsening process of a large number of Cu-rich droplets in Pb-rich matrix. We set 2 × 10^5^ droplets at the beginning. Their initial position is chosen randomly within the sample, and the initial number and size distribution of droplets is determined by the statistical data of a quenched Cu-Pb sample with an original composition of Cu-55 at % Pb. The samples were machined into a cylindrical shape of 10 mm in diameter and 20 mm in length. This sample was melted at 1050 °C and cooled at the rate of 0.74 °C/s, and then quenched at a temperature of 963 °C (10 °C below the binodal line in the phase diagram when the droplets have nucleated and grown to their required size.). The simulation of droplet coarsening is also set to start when the average temperature reaches 963 °C in the melt.

The collision of droplets depends on a precise estimation of the distance between them, therefore, a 3-D simulation has been adopted for the position and motion of droplets. The physical properties of droplets and liquid matrix were assumed to be dependent on the temperature of the grid they are located in. The properties of liquid Cu-Pb are shown in Table 1. The droplets are treated as spheres, having properties such as radius, positions, speed, and they know the grid where they are located in at the right moment. Having a fixed position in the sample at the right moment, the droplets are able to move in the temperature field by Marangoni motion and in the gravity field by Stokes motion.

The relative motion which leads to droplet collisions is assumed to be the different velocities of different-sized droplets because of thermocapillary effects and residual gravity. The droplets in immiscible alloys move relative to the matrix owing to various factors (gravity, natural convection, interfacial tension due to temperature, or concentration gradient.), leading to Stokes motion, Marangoni migration, and Brownian collisions. Our model simulates droplet growth by the diffusional growth, collision, and coalescence mechanism only, neglecting the process of nucleation and wetting. Here we use a method to eliminate the need to simulate nucleation, and use the statistical data of the initial drop-size distribution of samples quenched in the temperature range of the miscibility gap. In this initial data the average radius has grown to a size where Brownian motion can be neglected (the critical radius is generally around 1 μm). Interfacial tension is a spatial function around a fluid droplet [15,16], which can be influenced by very small particles located at the interface, such as impurities in metallic alloys or air bubbles. It should be mentioned that not all impurities have such an effect, only particles chosen properly have the feature of being located at the interface and act on the interfacial tension [17,18]. Instead of influencing the interfacial tension, most particles and impurities in metal immiscible alloys locate within one of the phases and serve as heterogeneous nucleation sites [19]. We considered that, in the Cu-Pb system, the effect of normal impurities on interfacial tension is negligible, and the methodology we used here neglected the process of nucleation.

The motion of a droplet can be predicted on the basis of Newton’s second law of motion by considering the various forces acting on the particle according to:

(7) $$\frac{d(g\rho_{p}{\vec{U}}_{p})}{dt}=\sum \vec{F}$$

where *g* denotes the gravitational acceleration and *ρ_p_* is the density of droplets, ${\vec{U}}_{p}$ is the instantaneous velocity of the droplet, and $\vec{F}$ represents the various forces acting on the body of the droplet.

The forces acting on the droplet include buoyancy force ${\vec{F}}_{buo}$, magnetic drag force ${\vec{F}}_{mag}$, viscosity drag force ${\vec{F}}_{drag}$, and interfacial gradient force ${\vec{F}}_{maran}$:

(8) $$\rho_{p}\frac{d{\vec{U}}_{P}}{dt}={\vec{F}}_{buo}+{\vec{F}}_{mag}+{\vec{F}}_{maran}+{\vec{F}}_{drag}$$

Buoyancy force ${\vec{F}}_{buo}$ depends on the density difference between droplets and the matrix:

(9) $${\vec{F}}_{buo}=g(\rho_{m}-\rho_{p})\vec{e_{x}}$$

where $\vec{e_{x}}$ is a unit vector in the direction of gravity, *ρ_m_* and *ρ_p_* are the density of Pb-rich matrix and Cu-rich particles, respectively. Since *ρ_p_* is lower than *ρ_m_*, buoyancy will force droplets to move opposite the gravity vector.

Drag force on spheres in conducting fluid under magnetic field is calculated by Reitz’s equation [20]:

(10) $${\vec{F}}_{mag}=-\frac{2}{5}\pi R^{3}\sigma {\vec{B}}^{2}{\vec{U}}_{p}\;\vec{B}//{\vec{U}}_{p}$$

(11) $${\vec{F}}_{mag}=-\frac{3}{5}\pi R^{3}\sigma \left(\sigma +3\sigma^{\prime }\right)/\left(2\sigma +\sigma^{\prime }\right){\vec{B}}^{2}{\vec{U}}_{p}\;\vec{B}⊥{\vec{U}}_{p}$$

Drag forces on single droplets moving in viscous fluid are described by the following equation [21]:

(12) $${\vec{F}}_{drag}=-2\pi R\eta_{m}({\vec{U}}_{p}-{\vec{U}}_{m})\frac{2\eta_{m}+3\eta_{p}}{\eta_{m}+\eta_{p}}$$

where *η_m_*, *η_p_* denote the dynamic viscosities of the matrix and droplets, respectively. ${\vec{U}}_{m}$ denotes the flow velocity of the matrix, and we assume here that ${\vec{U}}_{m}$ has the same value as $\vec{U}$.

Thermocapillary migration, also referred to as Marangoni migration, is the motion of droplets which occurs because of the gradients in surface tension. Surface tension is a function of composition and temperature, and gradients of concentration or temperature give rise to gradients of surface tension. Here we only consider the temperature gradient. The Marangoni motion is driven by the interfacial gradient force follows as [22]:

(13) $${\vec{F}}_{maran}=-\frac{4\pi R^{2}}{\left(1+\eta_{p}/\eta_{m})\cdot (2+\lambda_{p}/\lambda_{m}\right)}\cdot \frac{\partial \delta }{\partial T}\cdot \nabla T$$

where *λ_m_* and *λ_p_* are the thermal conductivity of the matrix and droplets, respectively. ∇*T* is the temperature gradient. *δ* is the interfacial tension. We called the force here the Marangoni force. From Equation (13), the Marangoni force is in the same direction with the temperature gradient.

Aboutalebi et al. [23] have simulated the trajectory of inclusion particles in a continuous casting process, and predicted the motion of particles on the basis of Newton’s second law, without considering the collision and growth behavior of these particles. In this work we use a similar method to track the motion of particles through Newton’s second law. However, in immiscible alloys, the growth of particles needs to be considered. For a moving droplet, the free convection and freely-developing concentration due to the interaction with the surrounding fluid could be simulated by imposing boundary conditions at the interface, like the model used by Yanxing Wang [24], whereas this method is so complicated that it is seldom used in the simulation of many droplets. In this work we use a simplified method to calculate the solute exchange between droplets and the surrounding matrix.

The diffusional growth can be described by the parabolic growth equation derived first by Zener [25]. Ratke further considers the effect of melt flow on this rate. The growth rate of droplets can be written as [21]:

(14) $$\frac{dR}{dt}=\frac{x_{Cu}^{}-x_{Cu}^{m}}{x_{Cu}^{p}-x_{Cu}^{m}}\frac{8}{\sqrt{3\pi }}D_{Cu}{}^{1/2}{\left(U_{p}-U_{m}\right)}_{}^{1/2}R^{-1/2}$$

where *D_Cu_* denotes the diffusion coefficient of Cu in Pb matrix. $x_{Cu}^{}$ is the concentration of Cu in the matrix surrounding the growing Cu-rich droplets. In this paper, we used the concentration in the grid where the droplet locates in. The mean matrix concentration has initially a value calculated according to the Cu content in alloy and in the droplets. $x_{Cu}^{m}$ and $x_{Cu}^{p}$ are the mole fractions of component Cu in equilibrium in the matrix and droplets, respectively. We assume here that ${\vec{U}}_{m}$ has the same value as $\vec{U}$ in the grid where the droplets are located. The loss of Cu concentration *R_Cu_* in Equation (6) can be written as:

(15) $$R_{Cu}=\sum \frac{4}{3}\pi \rho_{p}[\left(R+\frac{dR}{dt}\right){}^{3}-R^{3}]$$

In this discrete multi-particle approach, the size and position of each droplet is memorized, and they know the grid where they are located at the right moment. *R_Cu_*, calculated by Equation (15), is the summation of all droplets located in a grid, and serve as a source term in Equation (6). It should be mentioned that if a droplet crosses several grids, the value of *R_Cu_* is supposed to be divided and shared among these grids.

The thermophysical parameters used in this paper are listed in Table 1, in which T_k_ and T_c_ are the temperature in Kelvin and centigrade, respectively. Some parameters change according to temperature, and the moving speeds of spheres are calculated, respectively, according to the temperature of the grid it is located in. The temperature dependence of the interfacial tension, Resistivity, viscosity, as well as for the density and density difference of the coexisting phases for the liquid Cu-Pb is considered during the calculation of forces acting on them. We wrote the simulation codes by Matlab (Mathworks, Natick, MA, USA). To eliminate errors, the model has been checked from some simple cases, such as one or several spheres, which is convenient when comparing with mechanical calculations, and compared with the results of physical experiments. When we enhanced the number of droplets up to 2 × 10^5^, we compared the results with experiments with similar conditions.

## 4. Numerical Results and Discussion

### 4.1. Distribution of Temperature and Flow Fields

**Figure 1:**
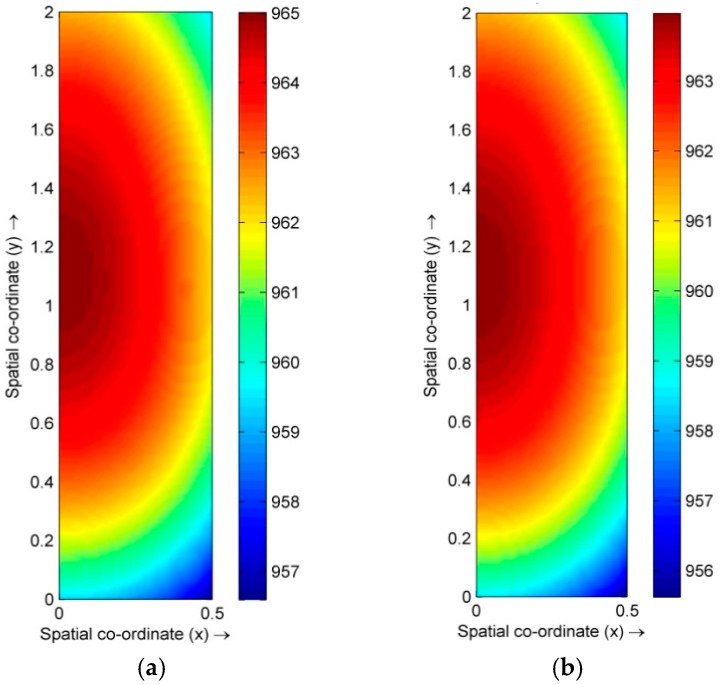
Temperature field in cooling Cu-Pb melts under 0 T and 12 T. (**a**) 0 T; and (**b**) 12 T.

**Figure 2:**
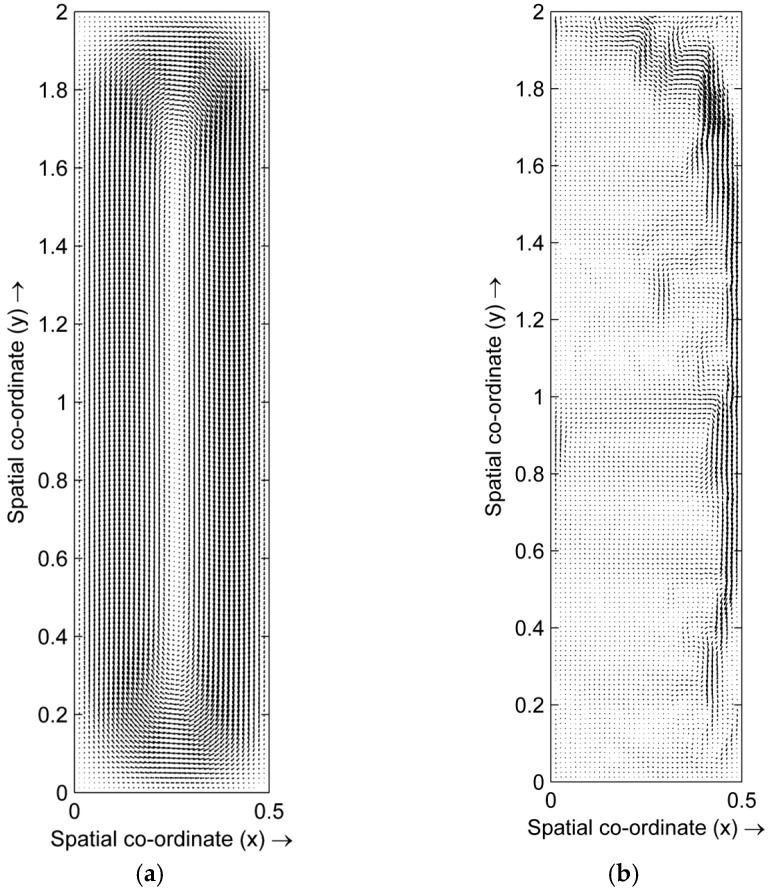
Flow velocity field in cooling Cu-Pb melts under 0 T and 12 T. (**a**) 0 T; and (**b**) 12 T.

We firstly consider the temperature field and the fluid velocity field of the melt. Figure 1 shows the temperature field in the Cu-Pb melt without the HMF and with a 12 T HMF, at the right moment of a mean temperature of 963 °C after cooling down from 1050 °C. The shape of the temperature field appears to be same in these two cases. Figure 2 shows the flow velocity field in the melt without the HMF and with a 12 T HMF. In the melt without the HMF, there is a clear circulating flow in the melt, whereas in the melt with the HMF the flow, in most regions, is suppressed and the main flow shifts to the regions near the side wall.

### 4.2. Distribution of Cu-Rich Droplets

**Figure 3:**
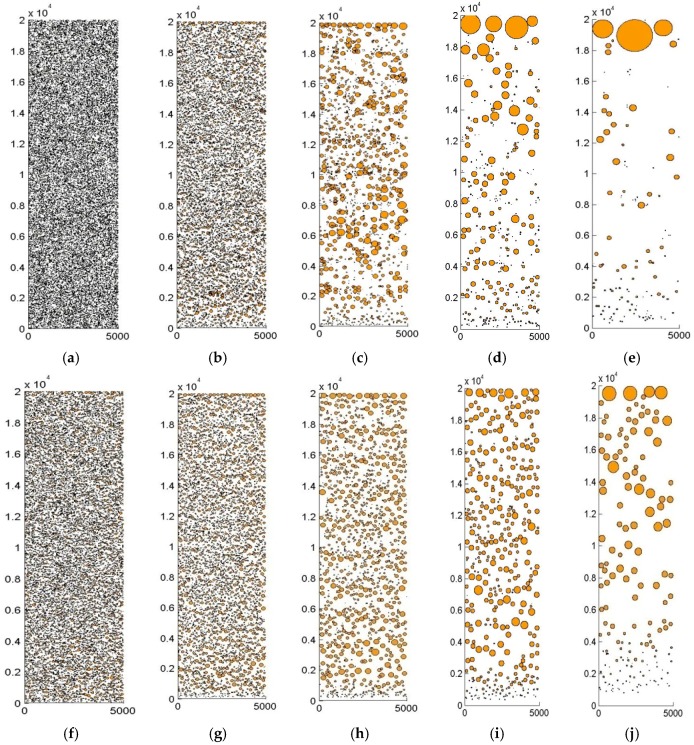
Spatial microstructure evolution with and without HMF. (**a**) 0 T, 0 s; (**b**) 0 T, 0.1 s; (**c**) 0 T, 0.2 s; (**d**) 0 T, 0.3 s; (**e**) 0 T, 0.5 s; (**f**) 12 T, 0.5 s; (**g**) 12 T, 1 s; (**h**) 12 T, 2 s; (**i**) 12 T, 3 s; and (**j**) 12 T, 6 s.

In the previous simulation work of immiscible alloy [9,10,26,27], the droplet population was either calculated as one group or divided into various size classes, the visualization of plenty of droplets in a real, continuously-cooling process is not convenient. In this paper, we allow all thermophysical properties to vary with temperature and time during the cooling process. Meanwhile the model also memorizes the position and velocity of each droplet, which provide a way to visualize the spatial distribution of droplets during the whole coarsening process. Figure 3 shows the evolution of the spatial distribution of Cu-rich droplets with and without the HMF. There is a dramatic coarsening rate in the sample without the HMF, which leads to a significant macro segregation occurring in 0.3 s, whereas a similar segregation occurs in 6 s with the HMF. The HMF has the effect of retarding the coarsening of Cu-rich droplets. In two samples of similar segregation degree, such as 0.3 s in the 0 T case and 6 s in the 12 T case, the number of very small droplets in the former appear to be larger than that in the latter, which means the anti-coagulation effect of the HMF is more effective on larger droplets and less effective on very small droplets.

A droplet move in the matrix with temperature gradient will move to the region of higher temperature under Marangoni force. This force is relatively small compared with gravity, hence, Marangoni migration is hardly observed in the results.

**Figure 4:**
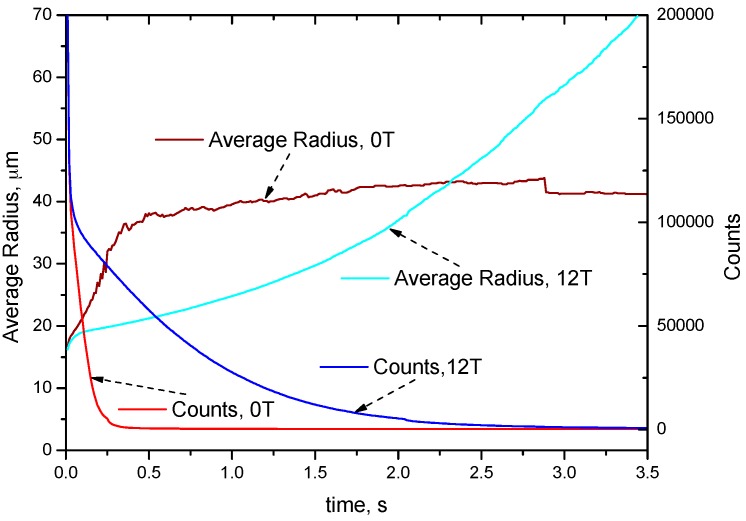
Average radius and number of droplets as a function of time.

Figure 4 shows the development of average radius and number of droplets in the samples with and without the HMF. In the sample without the HMF, the number of droplet decreases dramatically in the stage before 0.5 s, whereas the decreasing rate is slowed by the HMF. The velocities of droplets are retarded in the magnetic field and, thus, the collision frequency is also reduced. In the sample without the HMF, the average radius of droplets grows to a range of 35~43 μm in less than 0.5 s and then remains this size, whereas it keeps raising up under the HMF. In the early stage without the HMF, the fast decreasing rate in the droplet number implies that collision is a primary cause of coarsening. In the later stage, there are few droplets surviving after 0.5 s, and they have a low collision frequency due to the long distance between them, hence, their sizes grow slowly due to diffusional growth. On the contrary, the collision frequency is low under HMF, and coarsening is due to all the mechanisms including diffusional growth, Stokes coagulation, and Marangoni coagulation. The above inference could be confirmed by comparing the results with the previous simulation work of Guo and Liu [26,27], in which the different coarsening mode condition in immiscible alloys are analyzed respectively. The development of droplet radius of 0 T case in Figure 4 follow a similar trend with the diffusional growth mode as in [26,27]. The development of the droplet radius of the 12 T case shows a similar trend with the lines in [26,27] containing all the effects of diffusional growth, Stokes coagulation, and Marangoni coagulation.

**Figure 5:**
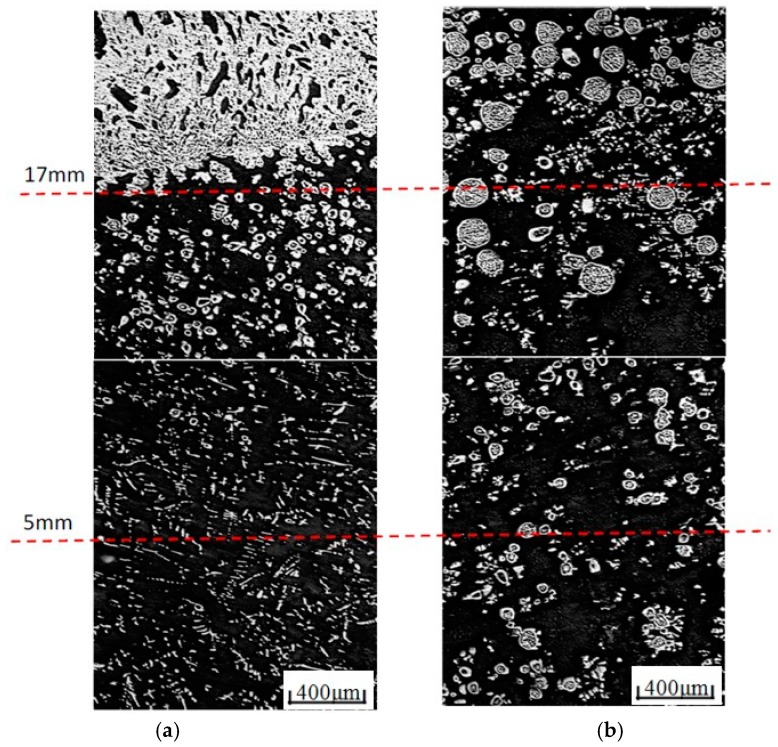
Solidified structure of Cu-55 at % Pb alloy, and the dotted line shows the distance from the bottom. (**a**) 0 T; and (**b**) 12 T.

It is difficult to make a direct comparison between experiments and simulations because accurate values of the heating and cooling times are not available. Moreover, the magnitude of the temperature gradient varies with position and with time during an experiment. Nevertheless, a rough comparison is performed here between the experimentally-observed microstructure and the results predicted by the simulations. The melting and solidification experiments are performed with liquid immiscible Cu-55 at % Pb hypermonotectic alloys under a 12 T HMF. The experimental microstructure is shown in Figure 5, in which the white round phase is the solidified droplets. The solidified droplets have different structure according to their size, and droplets with a radius larger than 40 μm show a net-shell type structure, with a net-like structure inside the spherical shell. Droplets smaller than 40 μm show an empty-shell type structure, which means the droplet consists of a Cu-rich spherical shell and the Pb-rich phase inside. It should be noted that the clusters of white-filled particles are not droplets, but Cu-rich dendrite. Without the HMF, the droplets will settle within the gravity field and the final state is an arrangement of two layers, with the lighter Cu liquid locating on the top. In the sample without the HMF, there are a large number of very small droplets, and most of them belong to the empty-shell type. The HMF appears to retard the coagulation of Cu-rich droplets, especially the larger ones. The amount of small empty-shell type droplets in a sample with the HMF is fewer than that without the HMF. This phenomenon is accordant with the simulation results in Figure 3.

**Figure 6:**
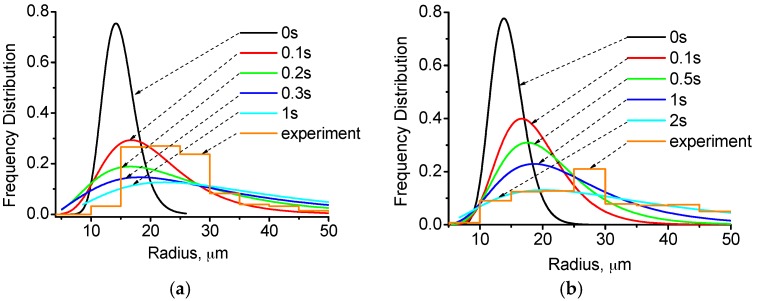

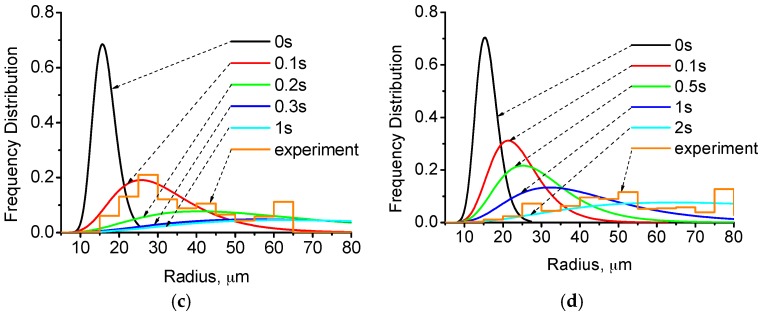
Evolution of a log-normal droplet radius distribution with time for 0 T case and 12 T case. (**a**) 0 T, in number density form; (**b**) 12 T, in number density form; (**c**) 0 T, in mass density form; and (**d**) 12 T, in mass density form.

Figure 6 shows the evolutions of droplet radius distribution in number frequency form and mass density form, respectively. It is demonstrated that the collision-coagulation caused a broader distribution. In the initial distribution, droplets have an average radius of R_n_ = 14 μm. Over the 1 s interval shown, there is an eight and four micron increase in the value of max radius frequency in the 0 T and 12 T case, respectively. The size distribution tends to spread to the region with larger values over time. The HMF affects the radius of the maximum frequency to rise slower. The measured drop-size distribution in the experiment is shown in Figure 6a,b in number density form by a histogram with a bin size of five microns. It is seen that the numerical simulation results with the HMF has a distribution that more closely represents the experimental results. Without HMF, the distribution frequency concentrates in the range of 15–40 μm, which is due to the capturing of plenty of large droplets by the Cu-rich segregation layer, and only a few droplets survive and stay in the matrix, most of them having a radius less than 40 μm. In the simulation results, large droplets keep their spherical shape instead of forming a layer, as a simplification.

It should be noted that even a small number of large droplets, which might appear negligible in number density form, can dramatically alter the evolution of a drop-size distribution. Smaller droplets have a much higher number density than larger ones and, hence, the figure of the number density frequency can hardly describe the size and number of large droplets. The relative mass fraction of the dispersed phase in the different drop-size categories is assessed and shown in Figure 6c,d. It is seen that the shift to larger droplets over time is more evident in terms of mass density rather than number density. The figures of mass density form also show the effect of the HMF to reduce the growth rate of droplets. The experimental result without the HMF is approximately accordant with the simulated distribution curve of 0.1 s, whereas the experimental curve with the HMF is accordant with the simulated curve of 2 s. As shown in Figure 4, the growth rate without the HMF tends to be slow after 0.3 s, the reason being that large droplets have floated up to the top, and the remaining small droplets have a small number and, hence, have a low collision frequency. In the experiment, the large droplets formed a segregation layer on the top, and the remaining small droplets grow slowly.

The whole time for liquid-liquid separation is 10.81 s from 963 °C to 955 °C. Below 955 °C the moving speed of Cu-rich solid particle is much slower than the liquid droplets, and the distribution of droplets in the end of the liquid-liquid separation is similar to the results of a solidified ingot [28]. A rough comparison between experiments and simulation shows that experimentally-observed drop-size distributions are not accordant to the final distribution at 10.81 s in the end of liquid-liquid separation, but accordant to the radius distribution at an early time predicted by the simulation, that is 0.3 s without the HMF and 2 s with the HMF. The discrepancy between the experiment and simulation is first caused by the existence of a segregation layer in the experiment, which captures some large droplets. A second explanation for this discrepancy is that the accuracy values for the quenching temperature and cooling rate in the experiment are not available. A third explanation for the discrepancy between simulation and experiment is that the forces calculated in this model do not reflect all the forces acting on a droplet. There are interactions among droplets which are complicated and need to be investigated in future research.

In the simulation work of Rogers and Davis [4], which used a population balance model to simulate a Zn-Bi system, the time evolution of the drop-size show a bimodal distribution. It is discussed that the second distribution of larger droplets developed perhaps due to droplet collisions. In this work, collisions are also considered, but the results only contain unimodal distribution. This discrepancy is perhaps due to that the coarsening stage being different. In the work of Rogers and Davis [4], the first peak of the bimodal distribution is less than 5 μm, the very small droplets have lower collision frequency; meanwhile, the larger droplets have faster collision frequencies and form a second peak of distribution. This work focus on the later stage of coarsening process, most droplets have larger size and they all take part in the collision coarsening process, which lead to a unimodal distribution of droplet radius.

**Figure 7:**
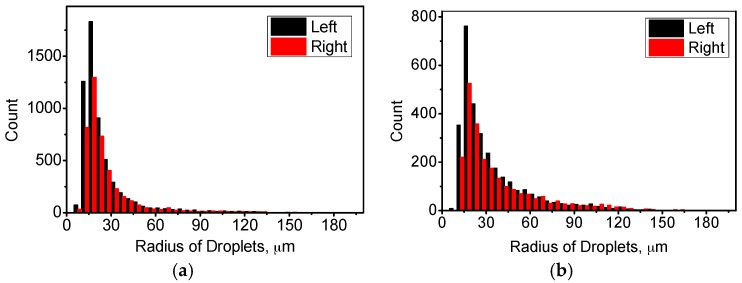
Comparison of radius distribution between left part and right part in case of (**a**) 0 T and (**b**) 12 T. (**a**) 0 T, 0.2 s; (**b**) 12 T, 2 s.

We assume that the gravity melt flow due to temperature difference has an effect on the motion of small droplets, whereas this effect is difficult to depict in the figure of spatial distribution. Therefore, we divide the sample into two parts, the left part and the right part. The left part means the center part of the sample, and the right part means the outer part of the sample. The melt flow is mainly upward in the left part and mainly downward in the right part (Figure 2). Figure 7 shows the distribution of droplet radius with and without the HMF, and the time is chosen to be 0.2 s in the 0 T case and 2 s in the 12 T case. The distribution tends to be different for these two parts, especially when the droplets are small. In the sample without the HMF, the amount of small droplets (smaller than 40 μm) in the left part tends to be higher than that in the right part. In the sample with the HMF, the amount is also higher in the left part for droplets smaller than 60 μm. The large droplets have larger velocities and higher collision frequency, and the influence of melt flow on large droplets is small and negligible.

**Figure 8:**
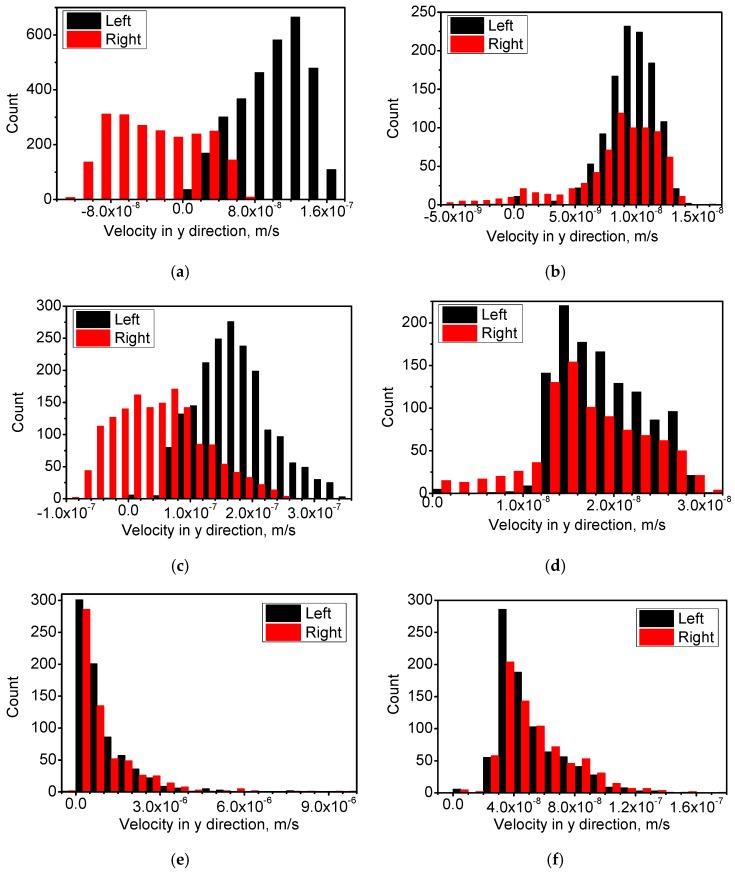
Comparison of migration velocity in the y direction of droplets in different size categories in both the left part and right part. (**a**) 0 T, 0.2 s, <20 μm; (**b**) 12 T, 2 s, <20 μm; (**c**) 0 T, 0.2 s, 20–40 μm; (**d**) 12 T, 2 s, 20–40 μm; (**e**) 0 T, 0.2 s, >40 μm; and (**f**) 12 T, 2 s, >40 μm.

Figure 8 shows the velocities of the droplets in the y direction (vertical upward) in different size categories. By comparing the distributions in the left and right parts in Figure 8, it is seen that the right part has a shift of the droplet velocity to higher values. Driven by the Stokes force, droplets move in the same direction with the melt flow in the left part, and in the opposite direction of the melt flow in the right part. A larger spread in the distribution in the right part implies that the relative velocities of the droplets are greater and, therefore, a higher rate of collision will be observed. When the radius is smaller than 40 μm, this shift of velocity to higher values is significant in the sample without the HMF. By comparing the distributions in the left and right parts in Figure 8, it is seen that the application of the HMF leads to a much slower velocity for the droplets. The velocity of the melt flow in the matrix is reduced under the HMF and, hence, it has a smaller influence on the velocity of the droplets.

The moving velocity of droplets in this work is much slower compared with the result of Jingjie Guo’s work [26,27], which is about the coarsening simulation of an Al-In system. The possible reason of this discrepancy is due to the different alloy system, and the Al-In system has a much larger density difference between droplets and matrix compared with the Cu-Pb system. The moving velocity in Figure 8 is also higher than in previous results by mechanical calculation of a single droplet in a Cu-Pb system [28]. The slow velocity in this work is partly due to the method to calculate the phase density. In the temperature region of miscibility gap, the minority Cu-rich phase and matrix Pb-rich phase (corresponding to the liquid L_1_ and L_2_ in the Cu-Pb phase diagram) have a much smaller density difference than pure Cu and pure Pb. In this paper, we try a new method to allow the density and all thermophysical properties to vary with temperature and time during the cooling process, which decreases the density difference and, hence, leads to the decrease of the droplet velocity. Compared with the experimental results, this method of calculation is more accordant to the real physical process. Melt flow in this model also changes the relative velocities of droplets, which leads to a different collision frequency. Melt flow has a significant influence especially at the first stage of coagulation when all droplets are small and their velocities are slower than the melt flow, and droplets tend to move along the direction of melt flow. Later, as droplets grow, Stokes motion takes on a key role. Large droplets move up, but fluid flow still pushes some small droplets, and changes the velocity and direction of them.

## 5. Conclusions

A numerical model is developed to predict the coarsening mode and the microstructural evolution under diffusional growth and collision coagulation during cooling of Cu-Pb immiscible alloys through the miscibility gap.

The effect of a HMF is considered in this simulation. In the sample with the HMF, the melt flow is inhibited in most regions. The moving velocity of liquid Cu-rich droplets is reduced by HMF, and the coagulation of droplets is retarded. The droplet number decrease slowly under HMF due to a low collision frequency and, thus, the sample has a much slower coarsening rate. There are a large number of dispersive droplets with a wide distribution in size. The migration of droplets is mainly due to Stokes motion. Marangoni motion is relatively small and negligible. The above phenomenon in the simulation results is accordant with the experiment of Cu-55 at % Pb alloy with a similar cooling condition.

The evolution of the droplet radius distribution also shows that the HMF reduces the coarsening rate. The size distribution tends to spread to the region of higher value over time, and the growth rate of the radius is slowed under the effect of the HMF.

The distribution of the velocity is very sensitive to the choice of droplet size, and the migration of small droplets with radii less than 40 μm tend to be affected significantly by the melt flow. The coarsening rate of the droplets is different between the inner part and outer part of the sample due to the different direction of the melt flow.

This study indicates that the coarsening process of immiscible alloys is affected by many factors. Future research should be performed regarding the melt flow, the interaction of droplets, and with a better accuracy for the values of temperature gradients and cooling rate.

## Figures and Tables

**Table 1:** Thermophysical parameters used for the simulation [13,14].

Parameters	Symbol	Pb-Rich Phase	Cu-Rich Phase
Density/g·cm^−3^	*ρ*	10.678 − 1.3174 × 10^−3^(T_c_ − 327)	8 − 0.801 × 10^−3^(T_c_ − 1083)
Resistivity/μΩ·m	*1/σ*	1.263 − 4.7 × 10^−4^(1000 − T_c_)	0.212 − 1.03 × 10^−4^(1200 − T_c_)
Viscosity/N·s·m^−2^	*η*	0.4636 × 10^−3^exp(1035.55/T_k_)	0.3009 × 10^−3^exp(366.83/T_k_)
Thermal conductivity/Wm^−1^·K^−1^	*λ*	26	165
Interfacial tension/Nm^−1^	δ	407 × 10^−3^(1 − T_k_/1268)^1.22^	
Diffusion coefficient	*D_Cu_*	420 × 10^−9^exp(−4850/T_k_)	
(Cu in Pb)/m^2^·s^−1^			
Specific heat capacity/J·K^−1^·kg^−1^	*c*	20.2 × (7.75 − 0.74 × 10^−3^ T_k_)	495
